# Green Manufacturing for Herbal Remedies with Advanced Pharmaceutical Technology

**DOI:** 10.3390/pharmaceutics15010188

**Published:** 2023-01-05

**Authors:** Martin Tegtmeier, Larissa Knierim, Axel Schmidt, Jochen Strube

**Affiliations:** Institute for Separation and Process Technology, Clausthal University of Technology, Leibnizstr. 15, 38678 Clausthal-Zellerfeld, Germany

**Keywords:** herbal remedies manufacturing, green technology, waste valorization, digital twins, quality by design, process analytical technology, autonomous operation

## Abstract

Herbal remedies are in most cases still manufactured with traditional equipment installations and processes. Innovative chemical process engineering methods such as modeling and process intensification with green technology could contribute to the economic and ecologic future of those botanicals. The integration of modern unit operations such as water-based pressurized hot water extraction and inline measurement devices for process analytical technology approaches in traditional extraction processes is exemplified. The regulatory concept is based on the quality-by-design demand for autonomous feed-based recipe operation with the aid of digital twins within advanced process control. This may include real-time release testing to the automatic cleaning of validation issues. Digitalization and Industry 4.0 methods, including machine learning and artificial intelligence, are capable of keeping natural product extraction manufacturing and can contribute significantly to the future of human health.

## 1. Introduction

**Figure 1:**
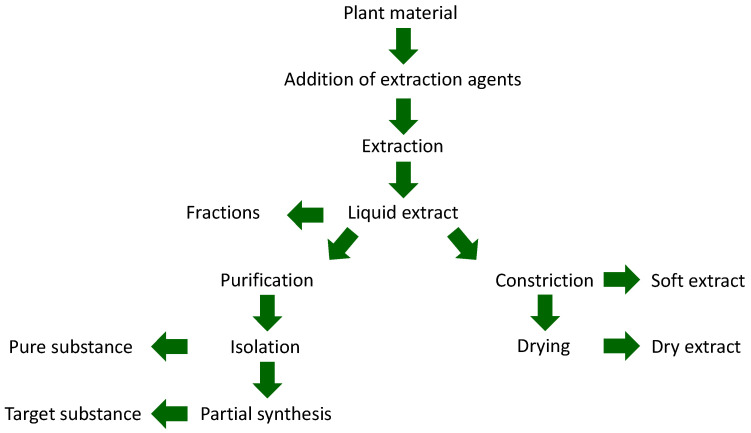
The process cascade for providing extracts and (modified) pure substances from plants.

**Figure 2:**
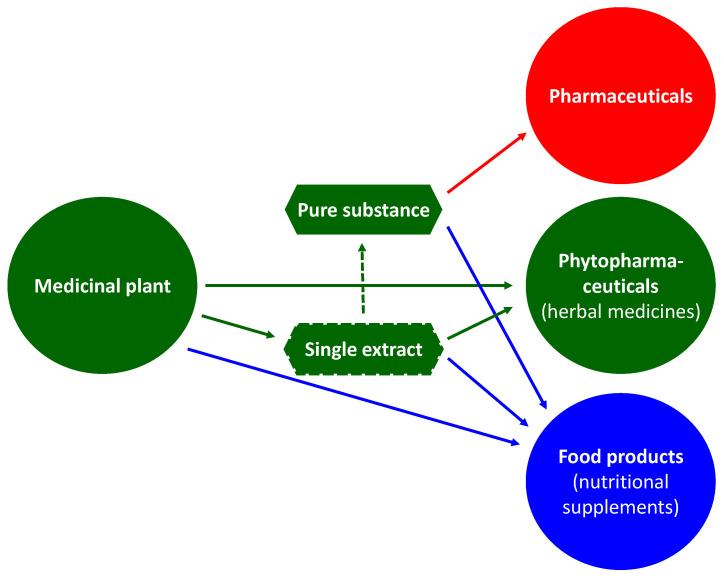
Plant parts can be used unmodified, as an extract or as an isolated pure substance for medicinal products, phytopharmaceuticals (herbal medicines), or foodstuffs (food supplements). Pharmaceuticals use pure substances as active ingredients, and phytopharmaceuticals (herbal medicines) contain plant parts or their extracts. Components of foodstuffs can be plant parts, extracts, or pure substances.

Medicinal plants and their importance in medicinal therapy are characterized among the lay public as medicinal tea and preparations derived from it, mostly as dragee, tablet, or juice. In fact, the spectrum of their use is much wider. While medicinal teas use the entire dried part of a medicinal plant, extractions can be used to focus on the relevant groups of constituents. Subsequently, there is the possibility of purification to obtain a pure substance, which is combined with the option for a final partial synthesis to arrive at the desired molecular structure. Examples include steroid hormones, corticoids, cardiac glycosides, artemisinin, and taxol. There is great interest in pure substances of natural origin, for example, substances with (supporting) antiviral effects or saponins (e.g., QS21 soap bark tree extract) as excipients for mRNA-based active ingredients [1,2]. Pharmaceuticals use these pure substances as active ingredients and functional agents, while phytopharmaceuticals (herbal medicines) contain plant parts or their extracts. Components of foods can be plant parts, extracts, or pure substances (Figure 1 and Figure 2) [3,4].

**Figure 3:**
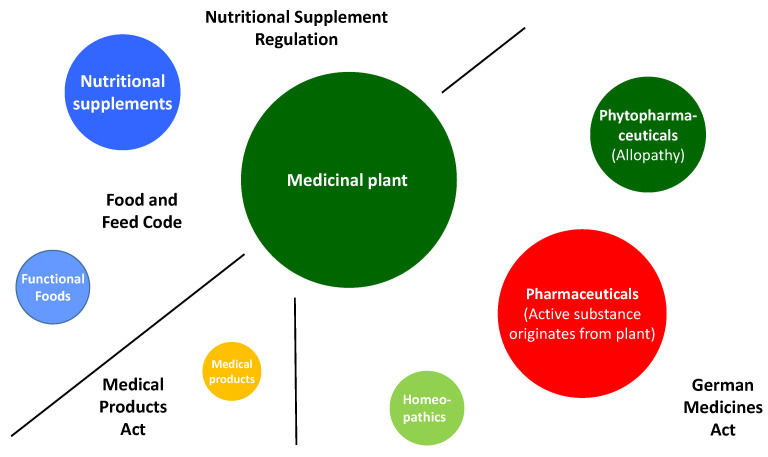
The use of plants from medicinal products to health products and the respective applicable legal norms in Germany.

The diversity is reflected not only in the way the ingredients of the respective plant are used but also in the regulatory framework for the use of the plant. Figure 3 shows the individual groups and the regulatory frameworks that apply to them in Germany: medicinal products (medicinal products with an active substance derived from a plant or multi-substance mixtures such as phytopharmaceuticals and homeopathics)—German legal norm: Medicinal Products Act (AMG); food supplements (NEM), functional food German legal norms: Food and Feed Code (LMFGB), Food Supplements Ordinance (NemV); Medical Device—German legal norm: Medical Devices Act (MPG).

The classic plant preparation is made either directly from the plant or with an extract produced from the plant. In both cases, mixtures of substances form the active ingredient. Phytopharmaceuticals, which follow the allopathic doctrine in pharmacology, represent the largest share of medicinal products. If one’s own studies on efficacy and safety have been carried out in the respective preparation, it can be approved in the European Union as “well-established use” (WEU). On the other hand, if a reference to the (pre)clinical results in one of the monographs of the Committee on Herbal Medicinal Products (HMPC) of the European Medicines Agency (EMA) is chosen, registration will follow [5]. In other regions, medicinal plants and the preparations derived from them may be legally categorized differently. For example, there are examples where a preparation is declared as a prescription drug in China, a pharmacy or over-the-counter drug in Europe, and a foodstuff in the USA.

In Germany, for example, important indications for phytopharmaceuticals are the treatment of colds with an increase in the body’s immunological defenses and gastrointestinal/bile complaints. Mild forms of depression or anxiety can be treated with St. John’s wort (*Hypericum perforatum*) and lavender (*Lavandula angustifolia*). Important niche indications of phytopharmaceuticals include preparations in gynecology for menopausal symptoms or the therapy of premenstrual syndrome, hemorrhoid remedies, urological drugs, and prostate and vein therapeutics [6].

A different therapeutic theory underlies homeopathic products, which, from a regulatory point of view, also belong to medicinal products. Medicinal plants or extracts obtained from them, such as mother tinctures, are also used for them. Food supplements, which have gained importance over the past two decades and are considered to be an interesting future option for health products based on herbs, fall into the legal area of foodstuffs. Herbal products have a niche character, which from a regulatory point of view fall into the group of medical products since their healing effect is not based on a pharmacological but a physical mechanism.

For several years, the demand for food supplements has been growing in many markets, e.g., in the field of dietary supplements and vitamins in the European as well as in the US market [7,8]. Compared to pharmaceuticals, market access for this type of food can be achieved much faster and with significantly less effort in Europe. Ideally, the selected plant already has a health claim from the European Food Safety Authority (EFSA), which certifies a positive effect on health when the plant is used. Examples of herbs and herbal extracts that are typically used as supplements in foods are; Guarana (*Paulinia cupana*) in drinks for extra energy and improved cognitive performance, St. John’s Wort (*Hypericum perforatum*) in drinks and cereals for a better mental balance, and Echinacea (*Echinacea purpurea*) in drinks for supporting the immune system.

Over the past three decades, the cost structure of phytopharmaceuticals has changed, especially in Central Europe. The discontinuation of reimbursement by most health insurance companies due to cost-saving measures led to significantly higher expenses for marketing and sales. At the same time, the expenditure for continuous regulatory work, such as ongoing stability studies, work on process validations, and updates of registration documentation have also risen sharply. Nagoya protocol guidelines, endocrine destructors, pyrrolizidine, and tropane alkaloids are new challenges regarding the old ones such as heavy metals and pesticide control [4]. The contaminant problem, especially, requires a lot of effort in the clarification and minimization of possible risks and subsequently constant additional investigations [9,10,11,12,13,14]. A corresponding passing on of the extra effort in the form of price increases is mostly ruled out since the market only allows this to a very limited extent. In this case, the size of the respective patient population in an indication group will determine whether the marketing still has prospects. If, on the other hand, there is a unique selling proposition in an indication area, moderate price adjustments will (still) be accepted by patients.

The economic situation described above also explains why hardly any new herbal medicinal products are developed to market maturity. Particularly in the case of new medicinal plants that have not yet been used in drugs, the cost of studies on efficacy and safety bears no relation to the potential return in subsequent marketing [15]. Thus, a paradox has developed in that, on one hand, the acceptance of and the desire for medicinal products from renewable plants is increasing more and more in the population while, on the other hand, the framework conditions are increasingly preventive in the fulfillment of these expectations. To what extent dietary supplements can represent an alternative path due to their lower share of approx. 5 to 10% of the cost of goods in the manufacturer’s selling prices in contrast to phytopharmaceuticals with up to 30% cost of goods remains questionable. So far, claims of the European Food Safety Authority on the respective health effect are needed, but these are not available yet [16].

Worldwide, a sales volume of approx. 130 billion € relating to 55% in medicinal plants can be assumed with a joint market share of approx. 50% for China and Japan [4,17].

**Figure 4:**
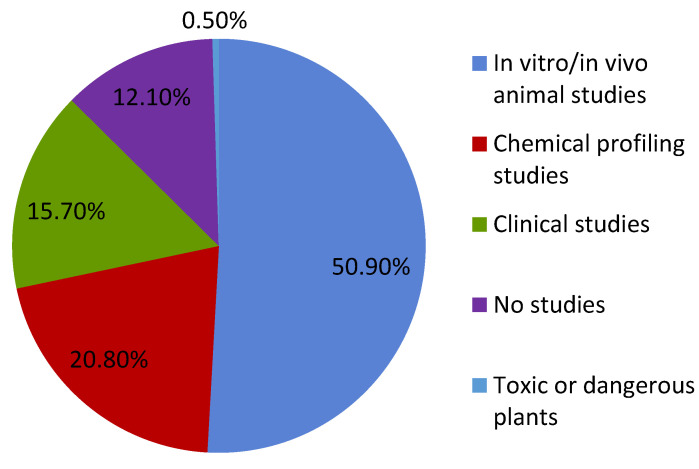
Status of studies on medicinal plants which investigated extracts and thus mixtures of substances and not pure substances according to [18].

Figure 4 gives an overview of the status of studies on medicinal plants, which investigated extracts and thus multi-substance mixtures and not pure substances. More than 50 % (e.g., *Echinacea purpurea* (L.) MOENCH) of the studies are preclinical, the results of which determine whether studies on patients (approx. 15%) (e.g., *Pelargonium radula* (Cav.) L’HERIT) and studies on the clarification of the ingredients (20%) (e.g., *Bovista plumbea* Pers.) take place. Only in a few cases (0.5%) did the studies concern toxically relevant plants (e.g., *Larrea mexicana* MORIC.), while about 12% of the plants in the western market (e.g., *Abrus precatorius* L.) have no published scientific studies on their properties [16].

## 2. Green Manufacturing for Herbal Remedies

The production of medicinal plants for the extraction of pure substances or as starting materials for phytopharmaceuticals is subject to strict regulations. For example, the use of herbicides and pesticides is prohibited, with very few exceptions. Mechanical weed removal is therefore used instead of herbicides. With the good agricultural and collection practice (GACP), which is a set of rules of the European Union, there are comprehensive and binding rules for cultivation, permanent cultivation, and wild collection [19,20,21,22]. GACP takes into account the specific conditions in the production and supply of plant starting material analogous to the requirements of the good manufacturing practice (GMP) guide Part 2 for the production of chemically synthesized active ingredients. Originally, GACP was a standard to be fulfilled under pharmaceutical law only within the European Union and shortly thereafter also in all member states of the area of validity of the European Pharmacopoeia. In the meantime, GACP is regarded as the essential standard and essential supplement to GMP regulations worldwide. Thus, GACP requirements must of course be taken into account in regulatory documentation and their implementation is subject to regulatory inspections and audits [6].

The cultivation of medicinal plants takes place as a special crop, which differs from the usual agriculture not just because of the GACP requirements. An essential feature is the significantly low land requirement, which results from the required quantities for plant-based active ingredients or extracts and can be easily understood [23]. The daily amounts of active ingredients per patient are in the mg range and extracts in the 100 mg range. Since only a few people need the medicines, manageable areas for the special crops are sufficient compared to the areas needed for food production (e.g., for grain). In Germany, medicinal plants are grown on about 12,000 hectares by about 750 companies, most of which have the structure of a small and medium enterprise (SME). However, more than 80% of the required amount of plant material is imported [4,17].

The extraction of medicinal plants is a sustainable production, which conserves resources through the careful use of renewable raw materials. In the entire process chain, from the plant to the drug, a consideration of CO_2_-based energy expenditures should be made.

The relevant process steps include the expenses of production (e.g., expenses for irrigation), harvesting (e.g., drying processes), extraction, and provision of pure substances (e.g., production of concentrates and dry extracts), as well as transport. In addition, however, activities in the surrounding area should also be considered, such as the provision of extraction agents and their fate after extraction is complete. Botanicals do have a higher global warming potential (GWP) CO_2,Eq_ factor of about 1000 times in comparison to basic chemicals; a figure of about 0.5 Mt GWP_CO2,Eq_ for 2030–2050 is expected. In the total balance, they account for an energy amount of about 1000 kWh/kg product [17].

Thus, a holistic view will lead to optimizations of existing production processes and to the establishment of new processes [24], which ideally require no or hardly any fossil resources for the provision of energy and chemicals such as extraction media [25]. If the regions for the provision of plant material also have production facilities for their (primary) processing in their vicinity, the cost of transport can also be reduced. Ideally, the drying of the plant material, which is essential for its shelf life during longer transports, can then be omitted by extracting the freshly harvested plants on site. The energy required for drying can be dispensed with and the transport volume is significantly reduced after extraction. In addition to essential contributions to achieving climate neutrality, there are also economic advantages for the regions where the plants are procured since the value is now added locally through (primary) processing of the plant material and the sale of higher-quality products becomes possible. A high acceptance of the implementation of the presented perspectives in the population can be assumed, but these ideas and their realization have to reach the people [23].

**Figure 5:**
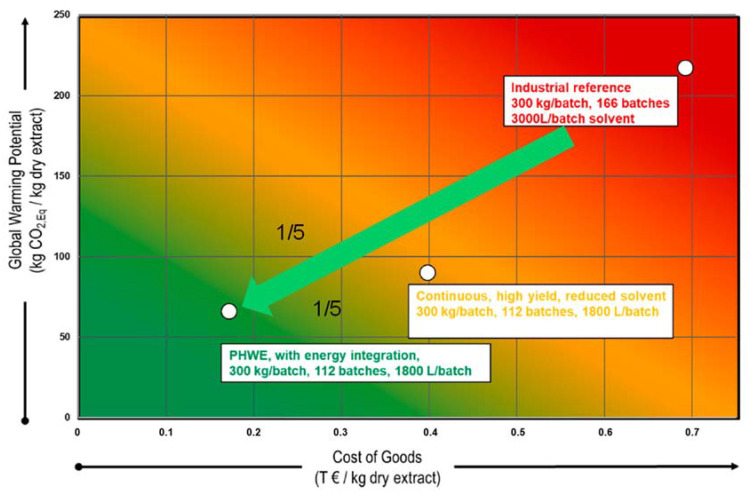
The influence of the extraction processes on the global warming potential and production costs [26].

If production costs are considered in relation to the GWP for different extraction processes, the advantages of hot water extraction outweigh the established use of organic extraction agents. Figure 5 also shows the importance of process optimization: by switching from batch to continuous operation and pressurized hot water extraction (PHWE) with energy integration, the costs of goods (COG) can be reduced from 700 € per kg of dry extract to less than 170 €, which corresponds to a cost reduction of about 1/5. In addition, GWP can be reduced from 230 to approx. 50 kg CO_2,Eq_ per kg of dry extract, which also corresponds to a reduction of approx. 1/5. A reduction of 1/5 in the cost of goods and 1/5 in GWP is achievable by any switch from batch to continuous operation with water-based processing as well as process optimization due to yield maximation [26].

## 3. Advanced Pharmaceutical Technology

When processing medicinal plants into drugs, the specifications of the European Pharmacopoeia must be taken into account [27]. Two categories exist for the definition of extracts: a pharmaceutical–technological consideration and a pharmacological classification. While the first category is binding for all extractions of plants, the second concerns only phytopharmaceuticals that contain extracts as active ingredients. Depending on the recognized state of scientific knowledge, the pharmacological classification differentiates between standardized extracts of medicinal plants for which the group of ingredients responsible for the effect has been defined, quantified extracts with specifications of relevant ingredient groups, and other extracts for which neither of the characteristics of the other two types of extracts is present.

The pharmaceutical–technological consideration defines the essential parameters for the extract types, starting with the process chain for liquid extracts, through further processing to the thick extract, to the final dry extract. An overview of the individual extract types is provided in Table 1 [27,28]. In addition, the essential process parameters of extraction are addressed, such as maceration or percolation as the basic process method, the extraction agent, and the significance of the respective process control.

The processing of freshly harvested plant material marks the beginning of the use of medicinal plants. In the Middle Ages and the beginning of the modern era, the first systematic extraction processes using freshly harvested plant material were developed in the context of tincture production. Questions regarding extending the shelf life led to the drying of the plant material, which had already been necessary before for the classical preparation of medicinal teas. Provided that the described restrictions on shelf life and transport do not exist, freshly harvested plant material can also be processed today. In most cases, the material is pressed and ethanol can be added for microbiological stabilization. If the water content in the plant material is sufficient, the water can be used as an extraction agent by heating the plant material. This process is referred to as “dry distillation”.

**Figure 6:**
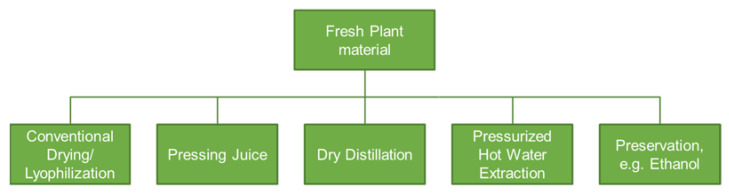
Options for processing freshly harvested plant material.

In Figure 6, additional optional processing options for freshly denatured plant material are presented, such as extraction with pressurized hot water or drying variants. In addition to conventional drying, lyophilization can also be useful for precious and sensitive ingredients, e.g., in flower constituents. Even today, fresh plant material should always be used for the production of mother tinctures for homeopathy.

Knowledge and experience from food production technologies show that the storage and transport stabilization of fresh plants can be estimated as low GWP and COGs-neutral and competitive. The continuous operation of extraction would need innovative logistic concepts and ideally fresh plant utilization because 70% of GWP contribution is related to drying. In relation to drying with about 0.24 €/kg at 10 mL/kg water steam with 2.78 kWh/kg [29], the first alternative is freezing/cooling with 0.015 €/kg at 648 kj/kg at 0.18 kWh/kg [29] or freeze-drying with about 0.017 €/kg at 2834.6 kJ/kg sublimation enthalpy with 6% equipment efficiency at 20.4 kWh/kg [30].

Batch to continuous operation since 2000 is an initiative demanded by the food and drug administration (FDA) for product quality improvement [31]. Since then, many studies have started to chemically synthesize active pharmaceutical ingredients (API) with the aid of flow chemistry [32] and then transferred them to industrialization [33]. Continuous manufacturing of pharmaceuticals is regarded as a product quality improvement due to batch harmonization and the reduction in batch lot failures [31]. Therefore, it starts industrialization with a MIT/Novartis cooperation [32] on synthetic active pharmaceutical ingredients; many studies have been published recently, trying to transfer such activities into biologics manufacturing [34,35,36,37,38,39].

The work on future process technologies for the extraction of plant parts is characterized by the principle of rationality rather than empiricism. New extraction processes can be developed into efficient and robust productions using the techniques of quality by design (QbD) and process analytical technologies (PAT) in a goal-oriented manner [16]. In this context, Industry 4.0 and digitalization in the form of a digital twin have an essential role [16,40]. Industry 4.0 aims at more automated processes by making machines more autonomous, able to communicate with each other and analyze large amounts of data. To achieve maximum efficiency in this regard, technologies such as simulation and digital twins, machine learning, and artificial intelligence are being used. After a systematic analysis of existing processes, it is also possible to optimize them according to the goals for new processes. With regard to the regulatory requirements for the registration documentation of medicinal products, it is important that Module 3 “Quality” is in the foreground when QbD and PAT are used. However, the effects on the processing of questions in modules 4 (“Non clinical study reports”) and 5 (“Clinical study reports”), for example, with regard to test and trial medications, must also be taken into account [41].

**Figure 7:**
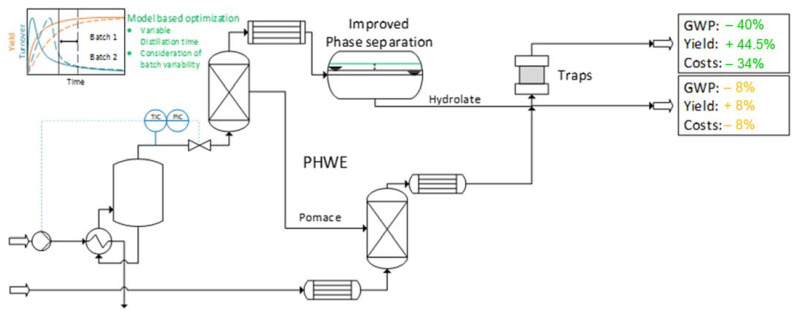
Process analytical technologies using the example of extraction with pressurized hot water extraction [17].

With the use of methods such as QbD in combination with PAT as well as digital twins and a model-based optimization, traditional plant extraction processes such as hydrodistillation can be optimized toward a reduction in the cost of goods as well as the reduction in the GWP of the process. In this case, the yield of the target components could be increased by a second extraction of the pomace of the hydrodistillation with a PHWE and following chromatographic adsorption on traps (s. Figure 7) [17].

However, the scope of optimization is determined, to a not-inconsiderable extent, by the regulatory framework. Improvements and thus changes in existing extraction processes for the provision of pure substances are likely to be rather easy to implement. In the case of European phytopharmaceuticals, new preclinical studies and new clinical studies are likely to be required for preparations with WEU approval. If the preparation has a registration with reference to a monograph of the HMPC, the cost of re-registration exceeds the savings achieved by optimizing the extraction.

**Figure 8:**
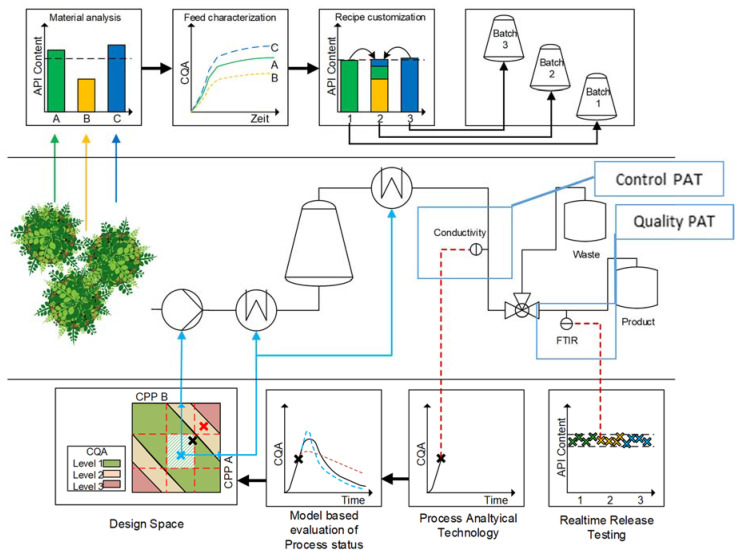
Process analytical technology strategy for control and quality-focused PAT to ensure advanced process control and real-time release testing [55].

Digital twins [42] are time-dependent dynamic process models, which are experimentally distinct, validated [43], and combined with process analytical technology [44] able to utilize real-time control. Even autonomous operation with feed-based recipe operation control is feasible. All methodological parts of such digital twins are already developed, available, and validated [43] for solid–liquid extraction performed as maceration, percolation, and PHWE [45,46] as well as hydrodistillation [47]. Digital twins are also developed and validated for the further processing of plant extracts, such as membrane technology [48,49], liquid–liquid extraction [50,51], crystallization [52,53], and precipitation [54]. For the use of digital twins, a feasible PAT strategy with an implemented partial least squares regression system has been developed [44,55]. In this context, an advanced process control strategy and a real-time release testing concept are also proposed and will be exemplified in further component systems (s. Figure 8) [40,56].

## 4. Advantages of Processes for the Provision of Pure Substances Taking into Account the Presented Optimizations Using the Example of Artemisinin and Taxol

Artemisinin serves as an anti-malaria drug, therefore, resource-efficient and economic processes for its production are needed. The process design was based on lab-scale experiments and afterward piloted on a mini-plant scale at the institute. A detailed economic feasibility study including a reference process as a benchmark for the lab-scale process and the pilot-scale process is given [51,57,58,59]. Relevant differences between the different scales are discussed. The details of the respective unit operations (solid–liquid extraction, liquid–liquid extraction, chromatography, and crystallization) are presented in dedicated articles. The study showed that even miniaturized lab-scale experiments can deliver data detailed enough for scale-up calculations on a theoretical basis. To our knowledge, a comparable systematic process design and piloting have never been performed by academia before. Green processing is reached with a significant GWP reduction due to solvent changes and solvent reductions by a factor of 4. COGs could be reduced by about 80% due to the optimization of the liquid–liquid extraction pre-purification which allows for the discarding of chromatographic purification steps completely. Yield improvements of about 40% with more efficient solid–liquid extractions and consistent process integration with the aid of process modeling lead to a total operating cost reduction of about 25%.

Thermodynamically consistent physical properties prediction methods such as COSMO-RS do support efficient solvent screening nowadays [60]. In this study, a systematic and model-based approach for a process development focusing on pressurized hot water extraction was investigated, considering the potential thermal degradation of high-value compounds. For the extraction of 10-deacetylbaccatin III (10-DAB) from yew as a representative test system, water at 120 °C provided the best compromise between extraction yield and thermal degradation. A yield of almost 100% with regard to the overall amount of 10-DAB was reached in only 20 min. Each experiment for model parameter determination was carried out with 1.9 g of plant material at a flow rate of 1 mL/min and an applied pressure of 11 bar. All experimental values were assessed by a physicochemical (rigorous) extraction model with experimental values and simulation results showing high conformity (R^2^ = 0.958). In order to demonstrate the usability of the extraction model and model parameter determination, a scale-up prediction was calculated. The scale-up experiments were predicted precisely and thus the model was validated. The experiments and the simulation results for a column with a volume of 104 mL and a mass of 22 g yew needles were consistent with the milli-scale used for the model parameter determination. A solvent reduction by a factor of 40 compared to the benchmark and by a factor of 10 lower investment costs resulted in 97 % COG savings, indicating the great importance of choosing the appropriate solvent in the first place. Green processing allows 99.5% of savings in raw material needle supply efforts [61,62].

## 5. The Use of Hot Water Extraction for the Preparation of Extracts of Phytopharmaceuticals Using the Example of Bearberry (*Arctostapylos uva-ursi* (L.) *SPRENG.*) with Arbutin as a Reference Substance [55]

Drugs containing extracts from the leaves of bearberry (*Arctostapylos uva-ursi* (L.) *SPRENG.*) are prescribed for the treatment of mild urinary tract infections and are a proven alternative to the use of antibiotics for this indication [63]. Hydroquinones are responsible for their efficacy, and arbutin has been established as the reference substance from the group of ingredients. The extracts belong to the class of “standardized extracts” according to the pharmacological classification of the European Pharmacopoeia. Based on the study situation, national WEU approvals could therefore be granted for some preparations in Europe. In the meantime, a monograph of the Committee on Herbal Medicinal Products of the European Medicines Agency also exists for bearberry, so traditional registrations for preparations with extracts of this medicinal plant are also possible with reference to the monograph [27,64].

Water-based processes totally exemplify the green extraction [65,66,67] approach perfectly. Additionally, they are kosher and halal-friendly, directly generally recognized as safe (GRAS), and therefore, represent ideal manufacturing technologies to meet market demands. A process sequence of PHWE and nanofiltration (NF) for concentration, followed by purification based on chlorophyll precipitation, liquid–liquid extraction for pre-purification and/or chromatography with final formulation by crystallization or direct lyophilization seems to be the most direct and logical manufacturing technology approach for the future, efficiently generating reliable product quality under all marked regulation demands. This could be systematically achieved by the quality-by-design (QbD) approach, which is demanded by regulatory authorities such as the FDA and EMA [68,69,70]. A central part of such innovative approval documentation is manufacturing operation robustness gained by the implementation of process analytical technologies [16,44].

In conclusion, a suitable approach may be to switch to, or at least to put more emphasis on standardized extracts, complete with efficacy studies and a new approval process supported by QbD-based process design, which enables process operation at its economical optimum.

This is beneficial, if not essential, for maintaining or regaining competitiveness in existing markets. Firstly, the batch variability of typical natural feedstock can be accounted for in the process design. Beyond that, it creates the technical basis for addressing increasing societal needs in the product development of innovative, plant-based antibiotics, and/or green and sustainable, resource-efficient manufacturing concepts with additional consumer benefits. The key role of plants in the medicinal and pharmaceutical fields for thousands of years is undisputed; however, it has had its difficulties. Nevertheless, even today, innovative molecules with therapeutic potential are quite often based on plants, which, in principle, have been known for decades or even centuries. Being able to break down complex natural mixtures into individual molecules or groups of molecules by advanced analytical tools allows for the characterization and testing for specific pharmaceutical/medicinal applications. Some of the best examples of successful applications in cancer and malaria treatments are taxol and artemisinin. Maybe the variety of plants and their broad scope of beneficial applications in healthcare might be exploited to find solutions to one of the biggest therapies needed by mankind.

## 6. Optimization of Hawthorn (*Craetaegus monogyna JACQ.*) Extraction [71]

Extracts from the leaves and flowers of hawthorn (*Craetaegus monogyna JACQ.*) belong to the pharmacological class of “other extracts” according to the specifications of the European Pharmacopoeia with hyperoside as the lead substance. Traditionally, hawthorn preparations are used for the treatment of mild cardiac complaints [27,72].

Traditionally used herbal medicines are deep in the consciousness of patients for the treatment of only minor diseases by self-medication. However, manufacturers of herbal medicinal products suffer from major problems such as increasing market pressure, e.g., from the food supplement sector, increasing regulations, and costs of production. Moreover, due to more stringent regulation and approval processes, innovation is hardly observed, and the methods used in process development are outdated. Therefore, this study aims to provide an approach based on modern process engineering concepts and including predictive process modeling and simulation for the extraction of traditional herbal medicines as complex extracts. The commonly used solvent-based percolation is critically assessed and compared to the so-called pressurized hot water extraction as a new possible alternative to replace organic solvents. In the study, a systematic process design for the extraction of hawthorn (*Crataegus monogyna JACQ.*) is shown. While for traditional percolation the solvent is optimized to a mixture of ethanol and water (70/30 *v*/*v*), the PHWE is run at a temperature of 90 °C. The extracts of various harvest batches are compared to a commercially available product based on a chromatographic fingerprint. For the first time, natural batch variability was successfully incorporated into the physicochemical process modeling concept. An economic feasibility study reveals that the PHWE is the best choice not only from a technical point of view but also from an economic aspect.

The study showed a systematic and model-based comparison of two different manufacturing methods for a traditionally used herbal extract. Both a percolation using a mixture of ethanol and water (70/30 *v*/*v*) as solvent as well as extraction with water at 90 °C show high productivity and yields. A high yield of the main flavonoid hyperoside as well as the desired range of the drug extraction ratio (DER) is reached. The chromatographic fingerprints revealed that all extracts were comparable to a commercially available product. The combination of experimental model parameter determination and rigorous process model is an efficient method of predictive process simulation, not only for the extraction of substances that are afterward purified to pharma-grade but also for the processing of traditionally used complex extracts. For the first time, natural batch variability was successfully incorporated into the physicochemical process modeling concept. These generated data sets are required by regulatory authorities demanding quality-by-design (QbD) and process analytical technology (PAT) approaches as modern tools with data-driven decisions documented for filing due to technological change, entering of markets of other countries, as well as changes in regional regulations and authority inquiry. An economic feasibility study showed that the PHWE can overcome the financial drawbacks of solvent storage and renewal efficiently, thereby justifying the higher investment costs for the necessary high-pressure equipment.

The consequent application of the process engineering toolbox of physical property calculations for solvent choice, miniaturized laboratory experiments for model parameter determination of all units, and efficient model validation with regards to accuracy and precision, as well as any process optimization based on cost modeling, allows for the gain in green processing benefits, the significant reduction in solvent consumption by up to a factor of 10, water-based processing technology, yield improvement to spare agricultural resources of about 90%, and COGs reduction of about 50% is realistic. Any change from batch to continuous operation results in significant operational and investment cost reductions by factors of about 5–10.

## 7. Case Study Standard Extract of Four Plant Sources

**Figure 9:**
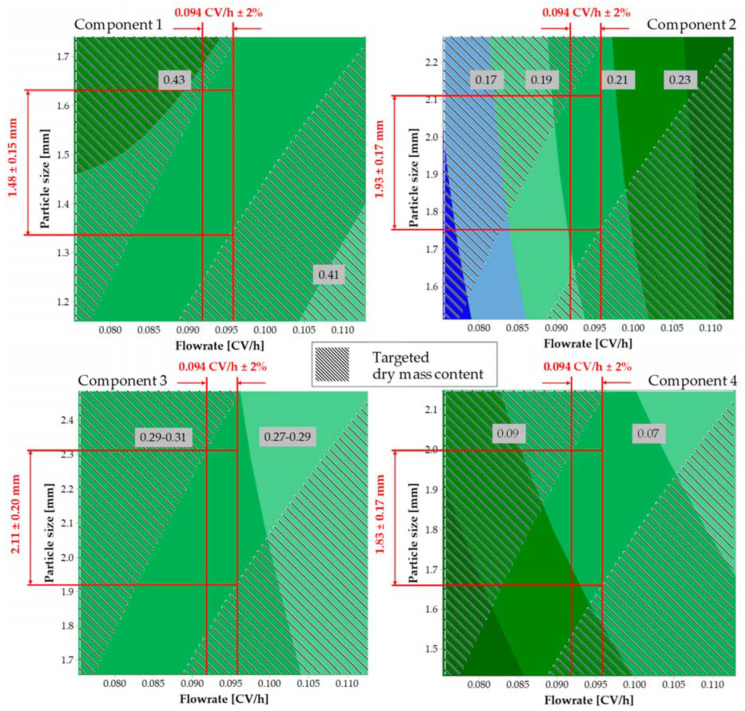
Design spaces for components 1–4 for varying flow rates and particle sizes [40].

A case study [40] exemplifies the quality-by-design approach to be generally applicable and of benefit. The main causes of batch deviations could be identified and highlighted that less than 5% were related to natural material variations An often taken prejudice could be data driven contradicted. Marker substances are used for each plant type, and it could be shown that equilibrium and kinetically controlled plant materials could be processed together, if intended, by finding common operation parameter design spaces. Predominantly, flow rate and extraction media volume ratio with regard to mean particle size distribution are the key parameters due to appropriate residence time and yield. In this case, even a main particle size deviation of about 10% was regarded as safe (Figure 9).

## 8. Conclusions

Innovative manufacturing technologies such as water-based processing based on pressurized hot water extraction followed by concentration with membrane technologies such as nanofiltration and ultrafiltration have general potential to gain the targets of climate neutrality and cost of goods savings to compete in worldwide markets successfully. Major reductions such as by a factor of five are feasible by using modern process design.

Drug quality assurance and improvement will be gained by applying the quality by design approach proposed and demanded by regulatory authorities. Such the transfer from classical batch-wise to continuous operation for a significant reduction of main resources needed is demonstrated.

Even feed-based recipe operations with simple proportional–integral–derivative-based process control and measurement devices such as pH, conductivity, and turbidity allow robust modern autonomous operation performances based on digital twins and process analytical technology. Such digitalization and Industry 4.0 methods, including machine learning and artificial intelligence, are capable of enabling traditional natural product extraction to compete with existing and future competition in markets.

The potential of fresh plant manufacturing is exemplified and the potential to gain new entities by efficacy studies for main therapies needed like anti-viral supporters, adjuvants and diabetes and heart insufficiency. Natural remedies, which are traditionally of great benefit, are thus enabled to continue to provide significant support to global health in the future within the context of recent increases in regulatory requirements for drug safety and improvements in manufacturing technology.

## Figures and Tables

**Table 1:** Extract types of the European Pharmacopoeia according to the pharmaceutical–technological classification.

Type of Extract	Classification of Extract According to the European Pharmacopoeia	
	**Designation**	**Latin Designation**
Fluid extracts	Liquid extracts	*Praeparationes fluidae ab extractione*
		*Extracta fluida*
	Tinctures	*Tinctura*
Concentrates	Soft extracts	*Extracta spissa*
	Oleoresins	*Oleoresina*
Dry extracts	Dry extracts	*Extracta sicca*

## Data Availability

Not applicable.

## Abbreviations

10-DAB	10-deacetalbaccatin
AMG	Medicinal Products Act
API	Active pharmaceutical ingredients
COG	Cost of Goods
DER	Drug Extract Ratio
EFSA	European Food Safety Authority
EMA	European Medicines Agency
FDA	Food and Drug Administration
GACP	Good Agricultural and Collection Practice
GMP	Good Manufacturing Practice
GWP	Global Warming Potential
HMPC	Herbal Medicinal Products
LMFGB	Food and Feed Code
MIT	Massachusetts Institute of Technology
MPG	Medical Devices Act
NEM	Food Supplements
NemV	Food Supplements Ordinance
NF	Nanofiltration
PAT	Process Analytical Technologies
PHWE	Pressurized Hot Water Extraction
QbD	Quality by Design
SME	Small and Medium Enterprise
WEU	Well-established use
